# Alleviation of Cartilage Destruction by Sinapic Acid in Experimental Osteoarthritis

**DOI:** 10.1155/2019/5689613

**Published:** 2019-02-26

**Authors:** Dawei Cai, Thomas W. Huff, Jun Liu, Tangbo Yuan, Zijian Wei, Jian Qin

**Affiliations:** ^1^Department of Orthopaedics, Sir Run Run Hospital, Nanjing Medical University, Nanjing, China; ^2^Department of Orthopaedics and Rehabilitation, Oregon Health & Science University, Portland, OR, USA

## Abstract

Sinapic acid (SA) modulates the nuclear factor-erythroid 2-related factor 2 (Nrf2) signaling pathway in chondrocytes. In order to test the hypothesis that SA is protective against the development of osteoarthritis (OA), primary mouse chondrocytes were treated* in vitro* with SA and the promoter transactivation activity of heme oxygenase 1 (HO-1), nuclear translocation of Nrf2, and protein expression of HO-1 were assayed. To test the hypothesis* in vivo*, a destabilization of the medial meniscus (DMM) model was used to induce OA in the knees of mice and SA was delivered orally to the experimental group. The chondrocytes were harvested for further analysis. The expression of HO-1 was similarly upregulated in cartilage from both the experimental mice and human chondrocytes from osteoarthritic knees. SA was found to enhance the promoter transactivation activity of heme oxygenase 1 (HO-1) and increase the expression of Nrf2 and HO-1 in primary chondrocytes. Histopathologic scores showed that the damage induced by the DMM model was significantly lower in the SA treatment group. The addition of a HO-1 inhibitor with SA did not show additional benefit over SA alone in terms of cartilage degradation or histopathologic scores. The expression of TNF-*α*, IL-1*β*, IL-6, MMP-1, MMP-3, MMP-13, ADAMTS4, and ADAMTS5 was significantly reduced both* in vitro* and* in vivo* by the presence of SA. Protein expressions of HO-1 and Nrf2 were substantially increased in knee cartilage of mice that received oral SA. Our results suggest that SA should be further explored as a preventative treatment for OA.

## 1. Introduction

Osteoarthritis (OA) is a common joint disease characterized by breakdown of articular cartilage. It is reported that about 18% of females and 10% of males aged over 60 years are affected [1]. Joint pain and stiffness decrease the quality of life for OA patients. Often joint replacement surgery is the best treatment option in late stages of the disease, when nonsurgical treatments are no longer effective. Progressive articular cartilage degradation is the hallmark of disease progression; thus inhibiting cartilage degradation slows or stops disease progression in OA [2]. Many potential therapeutic targets have been identified.

Nuclear factor (erythroid-derived 2)-like 2 (Nrf2) is one of these potential targets for treatment. Excessive oxidative stress leads to chondrocyte apoptosis in the progression of OA [3, 4]. Nrf2 is a transcription factor which regulates its downstream gene expression by controlling the antioxidant response elements (AREs) located in the promoter regions of its target genes, including antioxidative enzyme heme oxygenase 1 (HO-1). Nrf2 is involved in several degenerative diseases in multiple organs, and activation of Nrf2 is developing into a potential treatment for age-related diseases [5–8]. It has been reported that disruption of Nrf2 increases the vulnerability of age-related retinopathy, vacuolar leukoencephalopathy, and cardiomyopathy. HO-1 is a downstream protein of Nrf2. Upregulation of HO-1 has been shown to be protective against cartilage destruction in knee joints of mice in both posttraumatic and primary ageing models.

We have previously demonstrated that depletion of Nrf2 leads to rapid destruction of cartilage damage in two separate models of osteoarthritis [9]. This has led us to target Nrf2/HO1 for the treatment of OA. Sinapic acid (SA) is a naturally occurring hydroxycinnamic acid which can be extracted from plants like black mustard seeds. SA is considered a natural antioxidant [10–12].* In vivo* study has shown that oral administration of SA increases expression of Nrf2 and HO-1 in kidney, lung, and colon tissues in rats [13, 14]. SA has also been shown to inhibit the IL-1*β*-induced production of inducible nitric oxide synthase (iNOS), nitric oxide (NO), prostaglandin E2 (PGE2), and cyclooxygenase (Cox)-2 in rat chondrocytes [15]. Considering the antioxidative and potential anti-inflammatory effects of SA, we hypothesized that SA can have a protective effect on cartilage in an animal model of osteoarthritis via upregulation of Nrf2/HO-1 and downregulation of catabolic and proinflammatory genes.

## 2. Materials and Methods

### 2.1. Human Patients

All human cartilage samples were collected in Sir Run Run Hospital. Osteoarthritis cartilage was obtained from 6 patients (aged 68.67 ± 4.85 years) diagnosed with OA who underwent total knee arthroplasty (TKA), and cartilage in control group was collected from 6 patients (aged 27.67 ± 8.72 years) with trauma of the knee joint without OA or RA. This study was approved by the Ethics Committee of Sir Run Run Hospital and informed consent documents were signed by the donors.

### 2.2. Experimental OA and Treatment of SA

Wild-type C57BL/6N male mice (24.5g ± 2.4g) aged 8–10 weeks which were purchased from SLAC Laboratory (Shanghai, China) were used to assess the protective effect of SA on cartilage* in vivo*. The mice were housed in standard mouse cages (10 animals per cage) in specific pathogen free (SPF) conditions under a light-dark cycle of 12:12 h at 25 ± 2°C with standard mouse food (RM3; Special Dietary Systems) and water ad libitum. All the procedures were conducted in accordance with Nanjing Medical University Institutional Animal Care and Use Committee (IACUC) guidelines. Mice were anesthetized intraperitoneally with ketamine (90 *μ*g/gm body weight) and xylazine (25 *μ*g/gm body weight), and their left knees were prepared for DMM surgery. Osteoarthritis was induced via sectioning of the medial meniscotibial ligament, which anchors the medial meniscus (MM) to the tibial plateau, following fat pad dissection, with the right knee undergoing sham surgery in which the ligament was visualized but not transected. Displacement of the medial MM occurs after the surgery, which caused focused weight bearing in a smaller area, subsequently leading to the development of OA. Mice received SA (Sigma-Aldrich, St. Louis, MO, USA 10 mg/kg) suspended in a 10% aqueous solution of Tween 80 by gastric gavage every other day (10mg/kg) postoperatively. Control mice were given an equal volume of vehicle via gavage (10% Tween 80 solution) [16]. Tin protoporphyrin IX (SnPP, an inhibitor of HO-1; Sigma-Aldrich, Carnforth, UK 6 mg/kg) was administrated intraperitoneally twice a day from day 14 postoperatively [17, 18]. Knee joints were collected for histological assessment according to the OARSI score by a single researcher who was blinded for groups and treatments [9].

### 2.3. Cell Culture

Collagenase D was used to digest articular cartilage which was collected from knees of 5-day-old C57BL/6N mice as previously described [19]. Chondrocytes isolated from articular cartilage of one litter were seeded on a 10-cm dish at an initial density of 5 × 10^5^ cells per dish. After reaching 80% confluence, cells were detached and randomly plated in 6-well plates (10^5^/well), cultured in Ham's F-12 (DMEM/F12) which contains 100 *μ*g/ml streptomycin, 100 IU/ml penicillin, 5% FBS, and 2 mM L-glutamine. Only the first passage of PMCs was used in our assays. Cell counting kit-8 (CCK-8; Beyotime, Nanjing, China) was used to measure the cell viability. Chondrocytes were then treated with different doses of SA at different time intervals for further examination.

### 2.4. Quantitative Real-Time Polymerase Chain Reaction (qRT-PCR) and Western Blotting

Knee cartilage collected from mice was isolated using TRIzol reagent (Invitrogen) after homogenization for RNA extraction. Briefly, the cartilage which was snap-frozen in liquid nitrogen and stored at −80°C until RNA extraction was ground with a pestle and liquid nitrogen-chilled mortar followed by TRIzol extraction. Each experimental unit is a pool of 1-2 cartilage compartments. When pooling was performed, the experimental unit was regarded as one. Purity and yield of RNA were determined by NanoDrop ND-1000 spectrophotometer (Nanodrop Technologies, USA). cDNA was generated from mature mRNA with PrimeScript RT Master Mix (Takara Bio, Otsu, Japan) under provided protocol. Q-PCR was performed by a 7500 real-time PCR system using SYBR Premix Ex Taq reagents. Relative quantification was defined as 2-ΔΔCt method. Quantitative PCR was performed in triplicate for each sample. Primer sequences are shown in Table 1. Proteins lysed from cartilage from primary mouse chondrocytes and the cartilage tissue in knee joint were determined and normalized for western blot as previously described [19]. No pooling was performed for human articular cartilage. Each experimental unit for mice is a pool of 2 cartilage compartments. Nuclear extraction was according to the manufacturer's instructions of the nuclear protein extraction kit (Beyotime Biotech Inc., Nanjing, China). Anti-actin and anti-lamin B were obtained from Cell Signaling Technology (Beverly, MA, USA). Anti-Nrf2 and anti-HO-1 antibodies were supplied by Bioworld Technology (Nanjing, China). Densitometry of protein band was performed for western blot analysis. Bands were semiquantified by reverse image scanning densitometry with Photoshop (version 6.0; Adobe, San Jose, California).

### 2.5. Luciferase Assays

To analyze HO-1 transcriptional activity, HEK293 cells plated in 24-well plates were transfected with plasmids using Lipofectamine 2000. On the next day, the cells were treated with 3 ng/ml and 9 ng/ml of SA for 12 h. The relative luciferase activity was measured with a luciferase assay system (Promega, Madison, WI, USA). Data was obtained by removing the background luciferase activity, normalized to Renilla luciferase activity. All assays were repeated at least three times independently.

### 2.6. Statistical Analysis

All data were analyzed with GraphPad Prism 6.0 software and expressed as means ± standard deviation. Data following Gaussian distribution were further analyzed via Student's t tests, data without Gaussian distribution via Mann–Whitney U test. P < 0.05 was considered statistically significant.

## 3. Results

### 3.1. HO-1 Expression Is Upregulated in Osteoarthritis Cartilage

**Figure 1:**
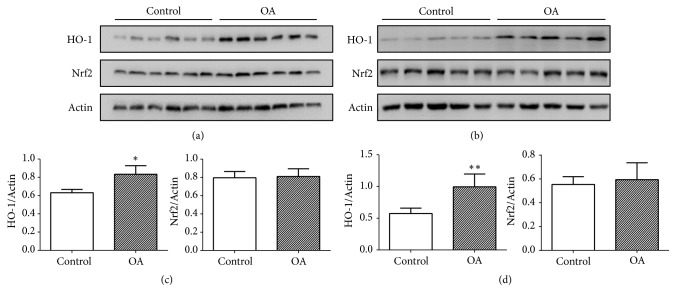
Heme oxygenase 1 (HO-1) expressions in cartilage. Human cartilage was separately collected from patients in control group and osteoarthritis (OA) group (n= 6 per group). Mice cartilage was obtained from knee of mice after DMM or sham surgery 4 weeks postoperatively (n= 5 per group). (a) HO-1 and Nrf2 of human cartilage were assayed by western blotting. (b) HO-1 and Nrf2 in cartilage from mice were assayed by western blotting. (c) Quantitative analysis of the expression level in human cartilage. *∗*p < 0.05. (d) Quantitative analysis of the expression level in cartilage from mice. *∗∗*p < 0.01.

Osteoarthritis cartilage was harvested from knee joints of human patients with OA and mice induced by sectioning of the medial meniscotibial ligament 4 weeks postoperatively. The control group was obtained from patients with trauma of the knee joint without OA or RA and mice undergoing sham surgery in which the ligament was visualized but not transected. Our result revealed that the level of HO-1 expression in the cartilage tissue from both human patients and mice with OA was much higher than the control group, while there was no significant different between the groups for Nrf2 expression (Figures 1(a)–1(d)).

### 3.2. SA Activates Nrf2 Signaling Pathway in Primary Mouse Chondrocytes

**Figure 2:**
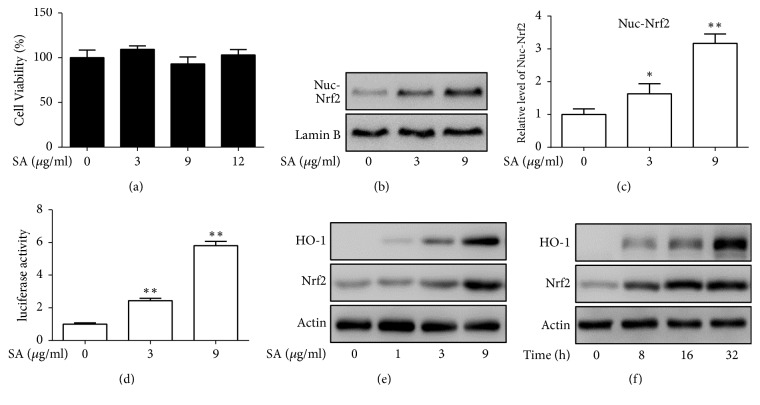
Activation of heme oxygenase-1 (HO-1) expression and nuclear factor-erythroid 2-related factor 2 (Nrf2) by SA in primary mouse chondrocytes. (a) Cell viability assay. Primary mouse chondrocytes were treated with different doses of SA for 12 h. A cell counting kit (WST-8) was used to measure the living cells. The assays were repeated three times independently. (b) Primary mouse chondrocytes were treated with SA (0, 3, 9 *μ*g/ml) for 12 h. The cell nuclear fractions of Nrf2 were tested by western blotting. (c) Quantification of nuclear Nrf2 from (b). *∗*p < 0.05, *∗∗*p < 0.01 compared with no-treatment control. (d) Transfected HEK293 cells were treated with 0 nM, 3*μ*M, and 9 *μ*g/ml of SA for 12 h and luciferase activity was measured. *∗∗*p<0.01 compared with no-treatment control. (e) Primary mouse chondrocytes were treated with various doses of SA (0, 1, 3, 9 *μ*g/ml) for 12 h. HO-1 and Nrf2 were assayed by western blotting. (f) Primary mouse chondrocytes were treated with SA (9 *μ*g/ml) for various times (0, 8, 16, 32 h), HO-1 and Nrf2 were assayed by western blotting.

In order to obtain nontoxic concentrations of SA used in further experiments, we analyzed the cytotoxicity in primary mouse chondrocytes. Cell viability did not decrease with increasing concentrations of SA up to 12 *μ*g/ml (Figure 2(a)). At these levels of concentration, our results showed that SA promoted nuclear translocation of Nrf2 and enhanced the promoter transactivation activity of HO-1 (Figures 2(b)–2(d)). We subsequently found protein expression of the HO-1, a Nrf2 downstream protein, was upregulated after treatment with SA (Figures 2(e) and 2(f)). These findings demonstrated that Nrf2/HO-1 signaling pathway was effectively activated by SA in primary chondrocytes.

### 3.3. SA Inhibits the IL-1*β*-Induced Expressions of MMPs and ADAMTSs in Primary Mouse Chondrocytes

**Figure 3:**
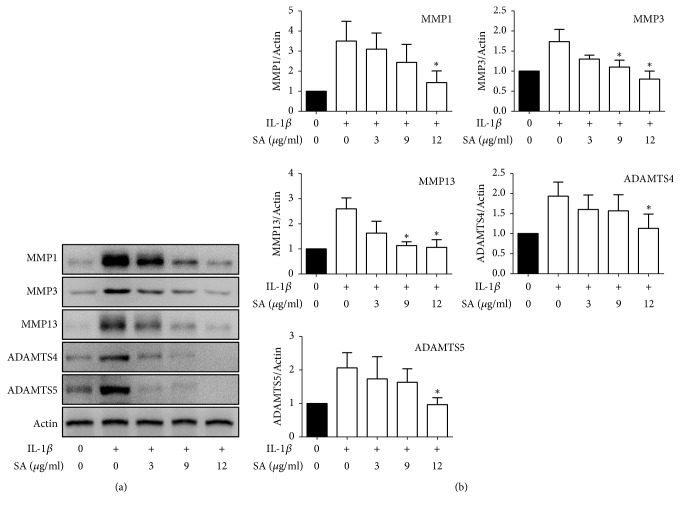
Effects of SA on IL-1*β*-induced MMPs and ADAMTS5 expression. Cells were pretreated with SA (3, 9, 12 *μ*g/ml) in the absence or presence of IL-1*β* (10 ng/ml) for 24 h. (a) Protein levels of MMPs and ADAMTSs were determined by western blot analysis. (b) Quantitative analysis of MMPs and ADAMTSs levels from (a). The bands are representative of three separate experiments. *∗*p < 0.05 compared with IL-1*β* treatment only.

MMPs and ADAMTS5 contributed to cartilage destruction during osteoarthritis. In order to test the therapeutic effects of SA on osteoarthritis, we examined the OA-related gene expression in chondrocytes pretreated with IL-1*β* for 24 hours (Figure 3) and found that SA decreased IL-1*β*-induced expressions of MMP-1, MMP-3, MMP-13, ADAMTS4, and ADAMTS5 in primary mouse chondrocytes in a dose-dependent fashion.

### 3.4. SA Protects against Cartilage Destruction of OA In Vivo

**Figure 4:**
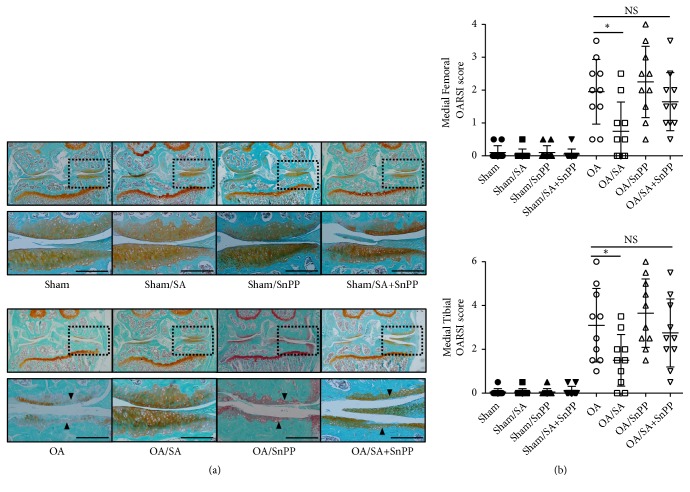
Histologic features and scoring for structural damage in mouse knee articular cartilage at 8 weeks following destabilization of the medial meniscus (DMM) model. OA was induced in C57BL/6N mice following surgical DMM, SA or vehicle was orally delivered every other day (10mg/kg) postoperatively. (a) Representative frontal sections (6 *μ*m) were stained with Safranin O/Fast Green (n=10 per group). Triangular symbol indicates the affected cartilage areas. Scale bars = 100 *μ* m. (b) OARSI scoring of OA. *∗*p < 0.05.

In order to determine whether SA slows the progression of OA* in vivo*, we delivered SA to the mice orally after surgical induction of OA. After eight weeks, histologic sections of knee joints were harvested and stained with Safranin O/Fast Green. We used OARSI scoring system to quantify the histopathological changes for cartilage destruction of knee joint. The result showed that scores for both femurs and tibias of SA-treated group were much lower than the control group (p < 0.05) (Figures 4(a) and 4(b)), which means oral administration of SA was protective against the progressive cartilage damage of osteoarthritis. In contrast, the cotreatment with SnPP and SA neither improved cartilage degradation nor changed the scores significantly in OA mice.

### 3.5. SA Reduces OA-Associated mRNA Expression

**Figure 5:**
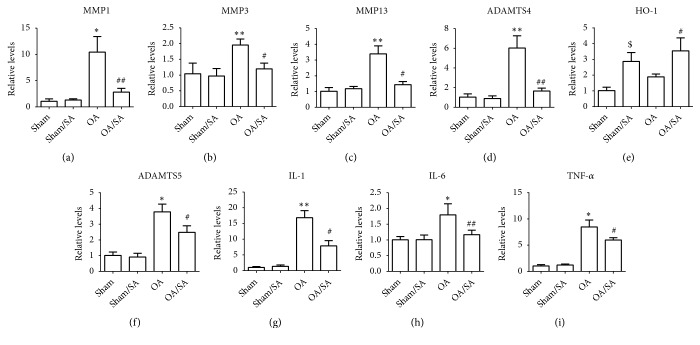
Relative expression of OA-related genes in cartilage from mice. (a-i) The OA-associated mRNA expression in cartilage from knee joints of mice was quantified by quantitative real-time polymerase chain reaction (qRT-PCR). *∗*p < 0.05, *∗∗*p < 0.01 compared with sham group. #p < 0.05, ##p < 0.01 compared with OA group. $p < 0.05 compared with sham group.

We collected cartilage of knee joint from mice and examined the levels of some OA-associated mRNAs by quantitative real-time polymerase chain reaction. Results showed that gene expression of TNF-*α*, IL-1*β*, IL-6, MMP-1, MMP-3, MMP-13, ADAMTS4, and ADAMTS5, which were all upregulated in cartilage of the control group, were significantly reduced in SA-treated group. In contrast, the expressions of HO-1 were increased in SA-treated group (Figures 5(a)–5(i)).

### 3.6. SA Promotes Expression of HO-1 and Nrf2 In Vivo

**Figure 6:**
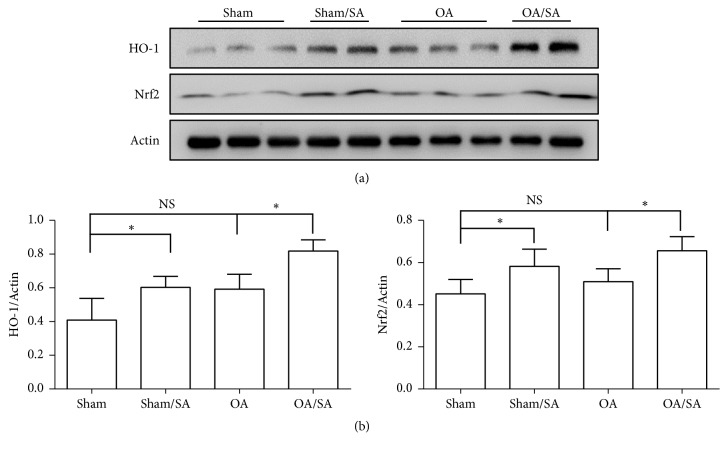
Effects of SA on the expression of HO-1 and Nrf2. (a) Representative western blot and (b) the expression ratio of HO-1 and Nrf2 to actin are presented (n=3; *∗*p < 0.05).

Expression of HO-1 and Nrf2 in knee cartilage from mice was tested by western blot to ascertain whether Nrf2/HO-1 was upregulated in the SA-treated group as was* in vitro*. Our data indicated that protein expressions of HO-1 and Nrf2 were substantially increased in knee cartilage of mice after systematic delivering of SA orally (Figures 6(a) and 6(b)), indicating that SA may protect against OA in mice in part by activation of Nrf2 signaling pathway.

## 4. Discussion

Oxidative stress has been shown to have a primary role in cartilage degradation in the progression of OA. Levels of several markers of oxidative stress change in OA patients. Altindag et al. found that total peroxide (TP) and lipid hydroperoxide in serum were significantly elevated in patients with knee osteoarthritis relative to patients without osteoarthritis [20]. Maneesh et al. observed that the levels of thiobarbituric acid reactive substances (TBARS) increased, while ascorbic acid, reduced glutathione (GSH), catalase, and glutathione peroxidase (GPx) levels all decreased in erythrocytes of subjects with osteoarthritis [21]. These results showed that the oxidants increased and antioxidants decreased in patients with osteoarthritis, indicating a relationship between oxidative stress and OA. Interestingly, not all antioxidants were inhibited in progression of OA. Activity of superoxide dismutase (SOD), an enzyme that catalyzes the disproportionation of superoxide, was found to be significantly increased in OA patients [21]. Fernandez et al. pointed out that heme oxygenase 1 (HO-1), which catalyzes the carbon monoxide, ferrous ion, and degradation of heme to bilirubin, was present in cartilage from both osteoarthritic and nonosteoarthritic patients [22]. Our study demonstrated that the level of HO-1 significantly increased in both patients with osteoarthritis and DMM induced OA mice relative to controls. The upregulation of HO-1 in cartilage may be regarded as a beneficial adaptive response to oxidative stress in chondrocytes during progression of OA. In fact, elevated expression of HO-1 was also observed in inflammatory arthritis. Kobayashi et al. reported that HO-1 expression was upregulated in synovial tissues of RA patients [23]. Maicas et al. also demonstrated that HO-1 was highly expressed in joint tissues of K/BxN serum‐induced rheumatoid arthritis mice [24].

Other studies have reported on the regulation of HO-1 in cartilage. Takada et al. found that lacking in Bach-1, a negative regulator of HO-1, enhanced expression of HO-1, which protected against cartilage destruction in knee joints of mice from posttraumatic and primary ageing models [25]. Nrf2 (nuclear factor (erythroid-derived 2)-like 2) is another regulator of HO-1 expression. It is known to control the expression of antioxidant proteins that are protective against oxidative damage. Nrf2 is normally kept in the cytoplasm and travels to the nucleus when under stressed conditions. It binds to the antioxidant response elements (AREs) located in promoter; one of its target genes is HO-1 [26]. Our previous study demonstrated that deletion of Nrf2 in mice leads to severe OA. Ansari reported that, with sinapic acid (SA), both Nrf2 and HO-1 were upregulated in kidney tissue from rats with cisplatin-induced nephrotoxicity [16]. Sinapic acid is an orally bioavailable phytochemical, adsorbed in the small intestine, which upregulates antioxidant levels in kidney, lung, and colon tissue when 10-20mg/kg body weight is given to rats and mice. It should follow that an antioxidant effect on arthritic cartilage by sinapic acid would be included in its systemic effects [10]. Our results showed that SA prompted Nrf2 translocation into the nucleus, which finally enhanced HO-1 expression in primary mouse chondrocytes. We next confirmed the protective effects of SA on cartilage* in vivo* in mice. In line with the results* in vitro*, levels of Nrf2/HO-1 in knee cartilage increased after the OA mice were treated with SA orally. In contrast, administration of SnPP, a HO-1 inhibitor, slightly reduced the protective effects of SA on cartilage, suggesting that SA may protect against oxidative damage via Nrf2/HO-1 in cartilage.

Although OA has long been classified as a noninflammatory arthropathy, more and more evidence has shown that inflammatory cytokines, chemokines, and other inflammatory mediators impact the osteoarthritis disease process throughout all stages [27, 28]. Studies in both human tissues and animal models have shown that articular chondrocytes are activated in osteoarthritis, leading to the disruption of homeostasis and aberrant expression of catabolic and proinflammatory genes [29, 30]. Our study indicated that increased expression of MMP-1, MMP-3, MMP-13, ADAMTS4, and ADAMTS5 in cartilage of OA mice was markedly suppressed by SA. These catabolic enzymes destroy aggrecan and collagen II, which are the two major components in the articular cartilage matrix. In our study, genetic analysis also revealed the upregulated expression of IL-1, IL-6, and TNF-*α* in osteoarthritis cartilage* in vivo* was suppressed by SA. Previous studies have suggested that the excessive production of these inflammatory cytokines in OA could inhibit matrix synthesis and promote the process of cartilage degradation [31]. In the study by Ansari et al., SA significantly suppressed the nuclear translocation of the p65 subunit of NF-*κ*B and DNA binding activity induced by cadmium in kidney tissues [32]. Huang et al. demonstrated that SA inhibited IL-1*β*-induced activation of MAPK signaling pathway [15]. HO-1, an inducible isoform of HO, degrades heme to carbon monoxide, biliverdin, and Fe2+, while carbon monoxide can suppress the synthesis of inflammatory mediators such as proinflammatory cytokines, nitric oxide, and prostaglandins. Related study revealed that overexpression of HO-1 inhibited the expression of adhesion molecules and generated interleukin-6 and nuclear factor-*κ*B activation in cultured human tracheal smooth muscle cells. HO-1 knockout and wild-type mice were used to prove that a general proinflammatory tendency is associated with HO-1 deficiency. These results indicate that the anti-inflammatory and anticatabolic effects of SA may contribute to a protective role for cartilage and the effect may be via HO-1.

Excessive oxidative stress and inflammation are involved in cartilage degradation in the progression of OA. In our study, SA upregulated Nrf2/HO-1 and reduced expression of catabolic and proinflammatory genes both* in vitro *and* in vivo*. This demonstrated a protective role for cartilage in the setting of OA. Although our study result showed that SA delivered orally to mice in the setting of knee OA helped prevent cartilage from degeneration, we have not determined whether SA is protective in prearthritic knees.

## 5. Conclusion

In summary, our study is, to our knowledge, the first to confirm the efficacy of SA in an animal model of OA. SA activated Nrf2/HO-1 signaling pathways and exerted potent anti-inflammatory effects in OA articular cartilage. These results suggest that SA may act as a potentially effective prevention option for OA.

## Figures and Tables

**Table 1:** Gene specific primer sequences qPCR.

Genes	qRT-PCR primers
IL-1*β*	Forward: ATGGCAGAAGTACCTAAGCTCGC
IL-1*β*	Reverse: ACACAAATTGCATGGTGAAGTCAGTT
IL-6	Forward: ACACACTGGTTCTGAGGGAC
IL-6	Reverse: TACCACAAGGTTGGCAGGTG
TNF-*α*	Forward: ATGAGCACAGAAAGCATGATCCGC
TNF-*α*	Reverse: CCAAAGTAGACCTGCCCGGACTC
MMP-1	Forward: GCCACAAAGTTGATGCAGTT
MMP-1	Reverse: GCAGTTGAACCAGCTATTAG
MMP-3	Forward: ATGAAAATGAAGGGTCTTCCGG
MMP-3	Forward: GCAGAAGCTCCATACCAGCA
MMP-13	Forward: ATGCATTCAGCTATCCTGGCCA
MMP-13	Reverse: AAGATTGCATTTCTCGGAGCCTG
ADAMTS4	Forward: ATGGCCTCAATCCATCCCAG
ADAMTS4	Reverse: AAGCAGGGTTGGAATCTTTGC
ADAMTS5	Forward: GGAGCGAGGCCATTTACAAC
ADAMTS5	Reverse: CGTAGACAAGGTAGCCCACTTT
ACTB	Forward: TGACGGGGTCACCCACACTGTGCCCATCTA
ACTB	Reverse: CTAGAAGCATTTGCGGTGGACGATGGAGGG
HO-1	Forward: ACATCGACAGCCCCACCAAGTTCAA
HO-1	Reverse: CTGACGAAGTGACGCCATCTGTGAG

IL: interleukin; TNF: tumor necrosis factor; MMP: matrix metalloproteinase; ADAMTS: a disintegrin and metalloproteinase with thrombospondin motif; qRT-PCR: quantitative real-time polymerase chain reaction; ACTB: Actin; HO: heme oxygenase.

## Data Availability

The data used to support the findings of this study are available from the corresponding author upon request.

## References

[1] Corti M. C., Rigon C. (2003). Epidemiology of osteoarthritis: Prevalence, risk factors and functional impact. *Aging Clinical and Experimental Research*.

[2] Garnero P., Piperno M., Gineyts E., Christgau S., Delmas P. D., Vignon E. (2001). Cross sectional evaluation of biochemical markers of bone, cartilage, and synovial tissue metabolism in patients with knee osteoarthritis: relations with disease activity and joint damage. *Annals of the Rheumatic Diseases*.

[3] Yudoh K., Nguyen V. T., Nakamura H., Hongo-Masuko K., Kato T., Nishioka K. (2005). Potential involvement of oxidative stress in cartilage senescence and development of osteoarthritis: oxidative stress induces chondrocyte telomere instability and downregulation of chondrocyte function. *Arthritis Research & Therapy*.

[4] Del Carlo Jr. M., Loeser R. F. (2003). Increased oxidative stress with aging reduces chondrocyte survival: correlation with intracellular glutathione levels. *Arthritis & Rheumatology*.

[5] Liu X., Lin X., Zhang S. (2018). Lycopene ameliorates oxidative stress in the aging chicken ovary via activation of Nrf2/HO-1 pathway. *Aging*.

[6] Silva-Palacios A., Ostolga-Chavarría M., Zazueta C., Königsberg M. (2018). Nrf2: Molecular and epigenetic regulation during aging. *Ageing Research Reviews*.

[7] Ray S., Corenblum M. J., Anandhan A. (2018). A role for Nrf2 expression in defining the aging of hippocampal neural stem cells. *Cell Transplantation*.

[8] Ahn B., Pharaoh G., Premkumar P. (2018). Nrf2 deficiency exacerbates age-related contractile dysfunction and loss of skeletal muscle mass. *Redox Biology*.

[9] Cai D., Yin S., Yang J., Jiang Q., Cao W. (2015). Histone deacetylase inhibition activates Nrf2 and protects against osteoarthritis. *Arthritis Research & Therapy*.

[10] Chen C. (2016). Sinapic acid and its derivatives as medicine in oxidative stress-induced diseases and aging. *Oxidative Medicine and Cellular Longevity*.

[11] Silambarasan T., Manivannan J., Krishna Priya M. (2014). Sinapic acid prevents hypertension and cardiovascular remodeling in pharmacological model of nitric oxide inhibited rats. *PLoS ONE*.

[12] Silambarasan T., Manivannan J., Priya M. K., Suganya N., Chatterjee S., Raja B. (2015). Sinapic acid protects heart against ischemia/reperfusion injury and H9c2 cardiomyoblast cells against oxidative stress. *Biochemical and Biophysical Research Communications*.

[13] Balaji C., Muthukumaran J., Nalini N. (2014). Chemopreventive effect of sinapic acid on 1,2-dimethylhydrazine-induced experimental rat colon carcinogenesis. *Human & Experimental Toxicology*.

[14] Raish M., Ahmad A., Ahmad Ansari M. (2018). Sinapic acid ameliorates bleomycin-induced lung fibrosis in rats. *Biomedicine & Pharmacotherapy*.

[15] Huang X., Pan Q., Mao Z. (2018). Sinapic acid inhibits the IL-1*β*-induced inflammation via MAPK downregulation in rat chondrocytes. *Inflammation*.

[16] Ansari M. A. (2017). Sinapic acid modulates Nrf2/HO-1 signaling pathway in cisplatin-induced nephrotoxicity in rats. *Biomedicine & Pharmacotherapy*.

[17] McDonnell C., Leánez S., Pol O. (2017). The inhibitory effects of cobalt protoporphyrin IX and cannabinoid 2 receptor agonists in type 2 diabetic mice. *International Journal of Molecular Sciences*.

[18] Nowis D., Bugajski M., Winiarska M. (2008). Zinc protoporphyrin IX, a heme oxygenase-1 inhibitor, demonstrates potent antitumor effects but is unable to potentiate antitumor effects of chemotherapeutics in mice. *BMC Cancer*.

[19] Gosset M., Berenbaum F., Thirion S., Jacques C. (2008). Primary culture and phenotyping of murine chondrocytes. *Nature Protocols*.

[20] Altindag O., Erel O., Aksoy N., Selek S., Celik H., Karaoglanoglu M. (2007). Increased oxidative stress and its relation with collagen metabolism in knee osteoarthritis. *Rheumatology International*.

[21] Maneesh M., Jayalekshmi H., Suma T., Chatterjee S., Chakrabarti A., Singh T. A. (2005). Evidence for oxidative stress in osteoarthritis. *Indian Journal of Clinical Biochemistry*.

[22] Fernández P., Guillén M. I., Gomar F., Alcaraz M. J. (2003). Expression of heme oxygenase-1 and regulation by cytokines in human osteoarthritic chondrocytes. *Biochemical Pharmacology*.

[23] Kobayashi H., Takeno M., Saito T. (2006). Regulatory role of heme oxygenase 1 in inflammation of rheumatoid arthritis. *Arthritis & Rheumatology*.

[24] Maicas N., Ferrándiz M. L., Brines R. (2011). Deficiency of Nrf2 accelerates the effector phase of arthritis and aggravates joint disease. *Antioxidants & Redox Signaling*.

[25] Ohta R., Tanaka N., Nakanishi K. (2012). Heme oxygenase-1 modulates degeneration of the intervertebral disc after puncture in Bach 1 deficient mice. *European Spine Journal*.

[26] Johnson J. A., Johnson D. A., Kraft A. D. (2008). The Nrf2-ARE pathway: an indicator and modulator of oxidative stress in neurodegeneration. *Annals of the New York Academy of Sciences*.

[27] Courties A., Gualillo O., Berenbaum F., Sellam J. (2015). Metabolic stress-induced joint inflammation and osteoarthritis. *Osteoarthritis and Cartilage*.

[28] Berenbaum F., Eymard F., Houard X. (2013). Osteoarthritis, inflammation and obesity. *Current Opinion in Rheumatology*.

[29] Iliopoulos D., Malizos K. N., Oikonomou P., Tsezou A. (2008). Integrative microRNA and proteomic approaches identify novel osteoarthritis genes and their collaborative metabolic and inflammatory networks. *PLoS ONE*.

[30] Puenpatom R. A., Victor T. W. (2009). Increased prevalence of metabolic syndrome in individuals with osteoarthritis: an analysis of NHANES III data. *Postgraduate Medical Journal*.

[31] Kapoor M., Martel-Pelletier J., Lajeunesse D., Pelletier J., Fahmi H. (2011). Role of proinflammatory cytokines in the pathophysiology of osteoarthritis. *Nature Reviews Rheumatology*.

[32] Ansari M. A., Raish M., Ahmad A. (2017). Sinapic acid ameliorate cadmium-induced nephrotoxicity: In vivo possible involvement of oxidative stress, apoptosis, and inflammation via NF-*κ*B downregulation. *Environmental Toxicology and Pharmacology*.

